# Supplementary material for: Evolutionary Dynamics of H5 Highly Pathogenic Avian Influenza Viruses (Clade 2.3.4.4B) Circulating in Bulgaria in 2019–2021
Source: Viruses. 2021 Oct 16;13(10):2086. doi: 10.3390/v13102086 (PMC8541051; doi:10.3390/v13102086)
Supplement: Supplementary file 1 [file viruses-13-02086-s001.zip › Supplementary Figures.pdf]

Supplementary Figures

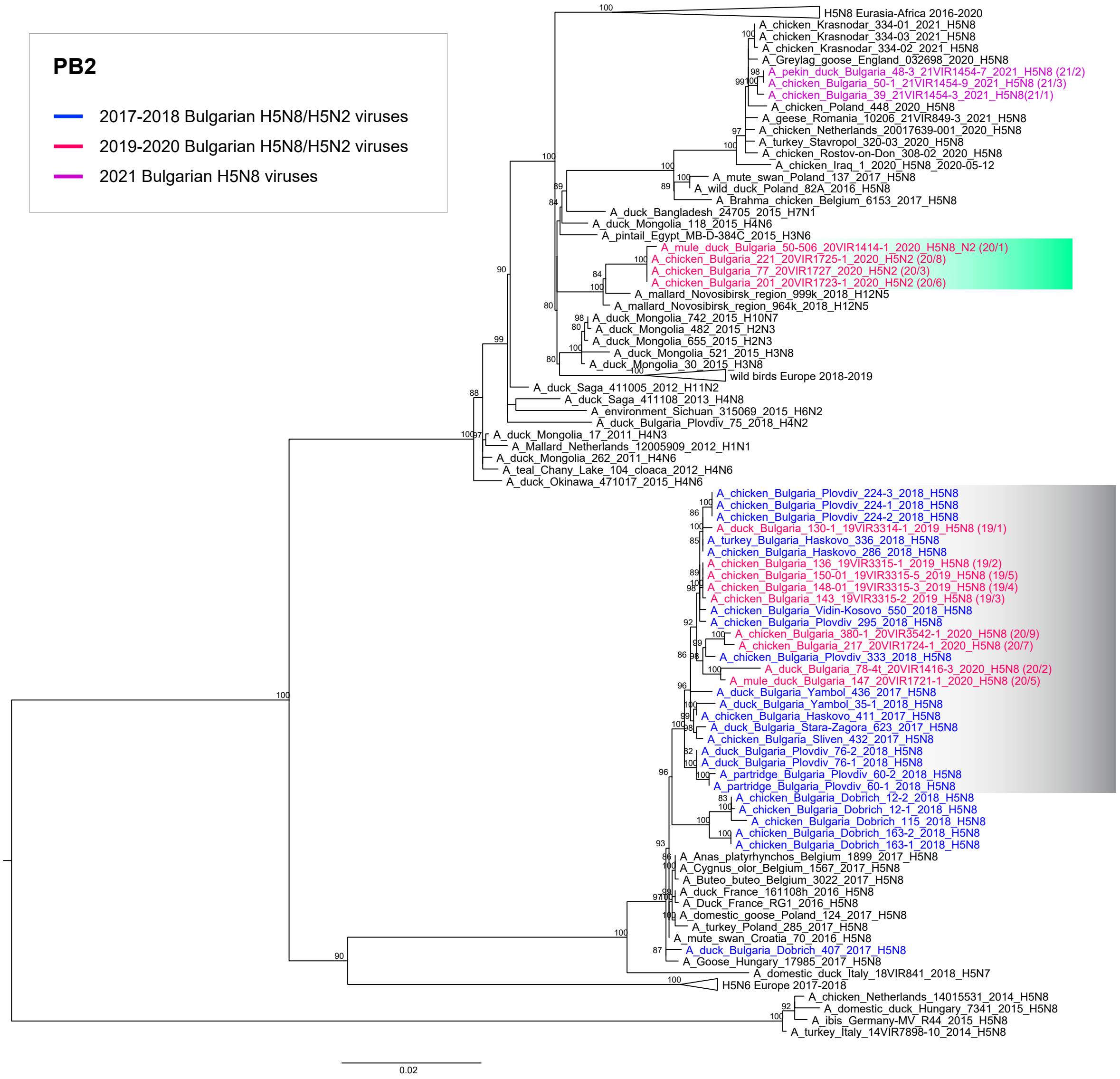

Supplementary Figure S1. Maximum Likelihood phylogenetic tree of the PB2 gene. The tree was inferred using IQTREE v1.6.6. The grey box shows the cluster of Bulgarian H5 (N8 and N2) viruses identified in 2019-2020 together with some previously reported 2018 H5N8-H5N2 detections; the light green box shows the cluster of 2020 Bulgarian H5N2 viruses. The viruses are coloured according to the years of collection. Ultrafast bootstrap supports higher than 80 are indicated next to the nodes.

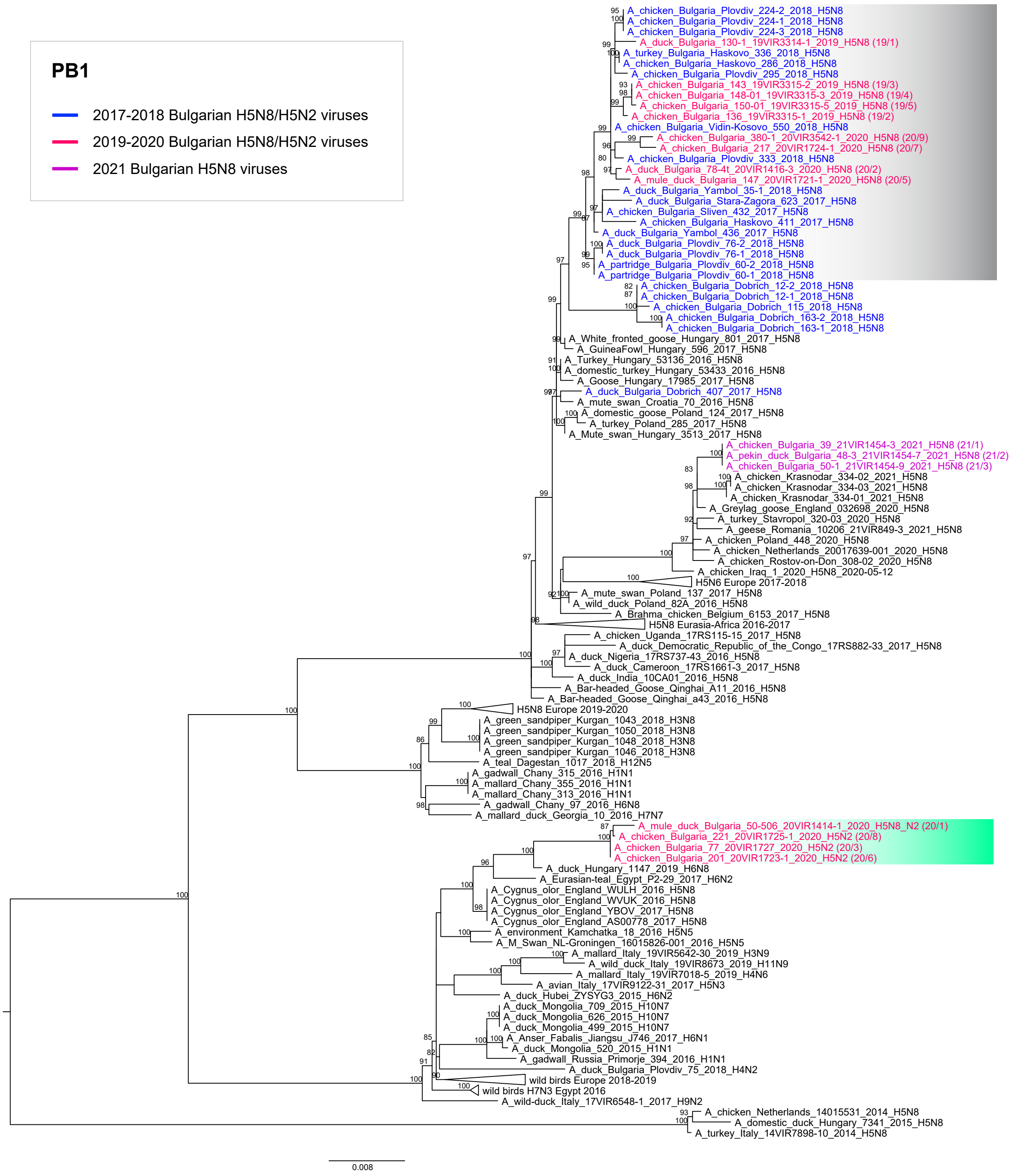

Supplementary Figure S2. Maximum Likelihood phylogenetic tree of the PB1 gene. The tree was inferred using IQTREE v1.6.6. The grey box shows the cluster of Bulgarian H5 (N8 and N2) viruses identified in 2019-2020 together with some previously reported 2018 H5N8-H5N2 detections; the light green box shows the cluster of 2020 Bulgarian H5N2 viruses. The viruses are coloured according to the years of collection. Ultrafast bootstrap supports higher than 80 are indicated next to the nodes.

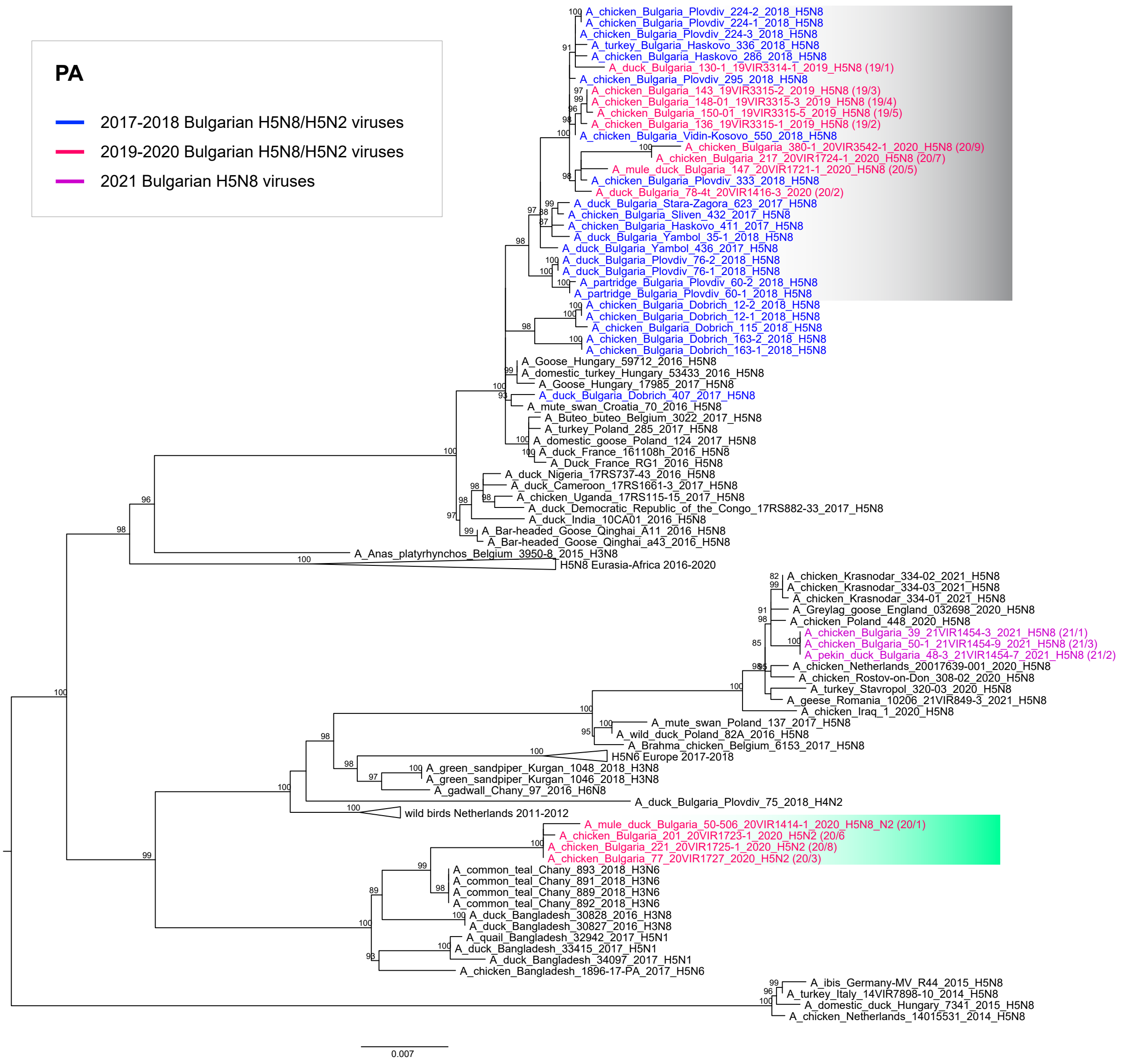

Supplementary Figure S3. Maximum Likelihood phylogenetic tree of the PA gene. The tree was inferred using IQTREE v1.6.6. The grey box shows the cluster of Bulgarian H5 (N8 and N2) viruses identified in 2019-2020 together with some previously reported 2018 H5N8-H5N2 detections; the light green box shows the cluster of 2020 Bulgarian H5N2 viruses. The viruses are coloured according to the years of collection. Ultrafast bootstrap supports higher than 80 are indicated next to the nodes.

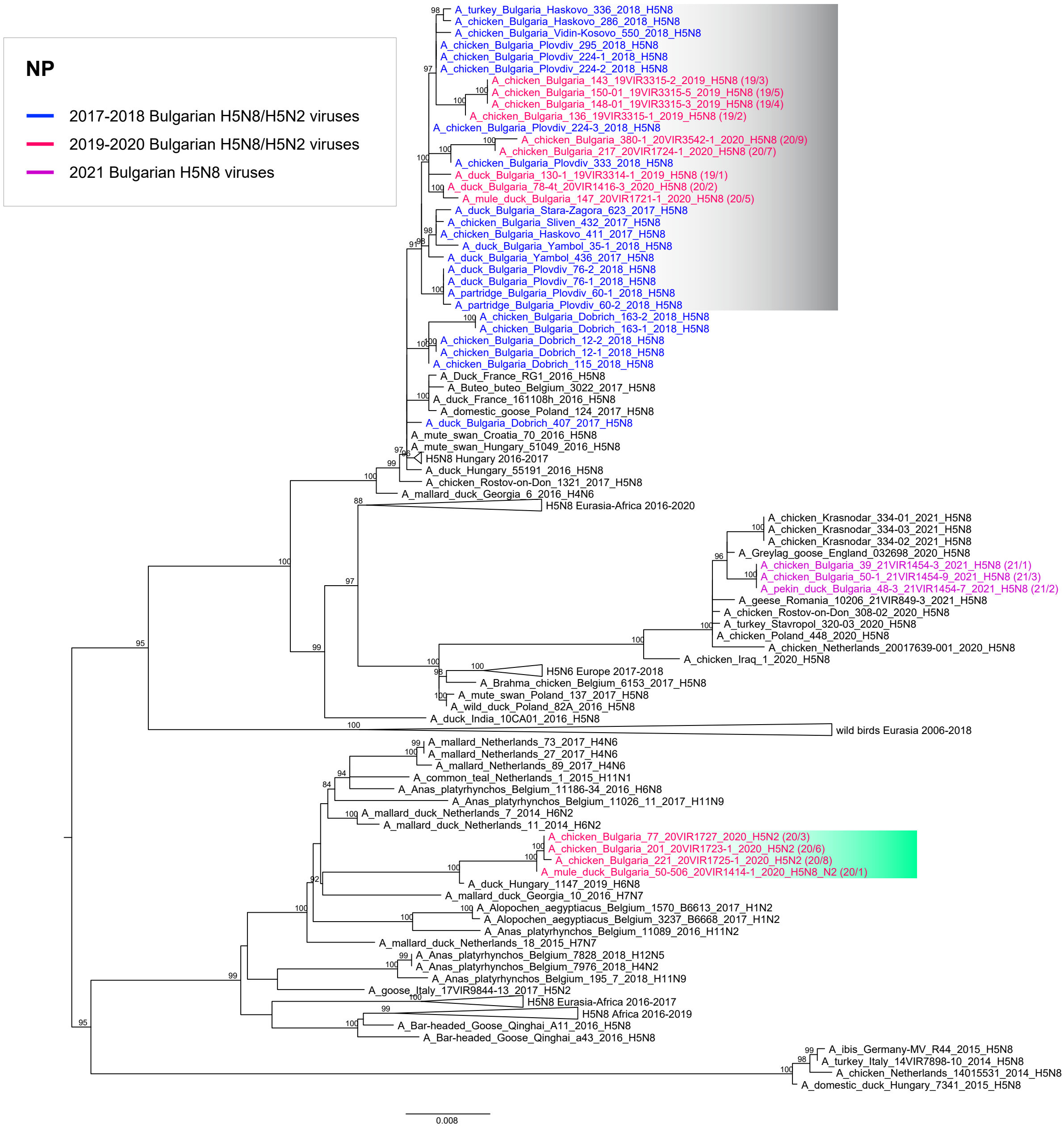

Supplementary Figure S4. Maximum Likelihood phylogenetic tree of the NP gene. The tree was inferred using IQTREE v1.6.6. The grey box shows the cluster of Bulgarian H5 (N8 and N2) viruses identified in 2019-2020 together with some previously reported 2018 H5N8-H5N2 detections; the light green box shows the cluster of 2020 Bulgarian H5N2 viruses. The viruses are coloured according to the years of collection. Ultrafast bootstrap supports higher than 80 are indicated next to the nodes.

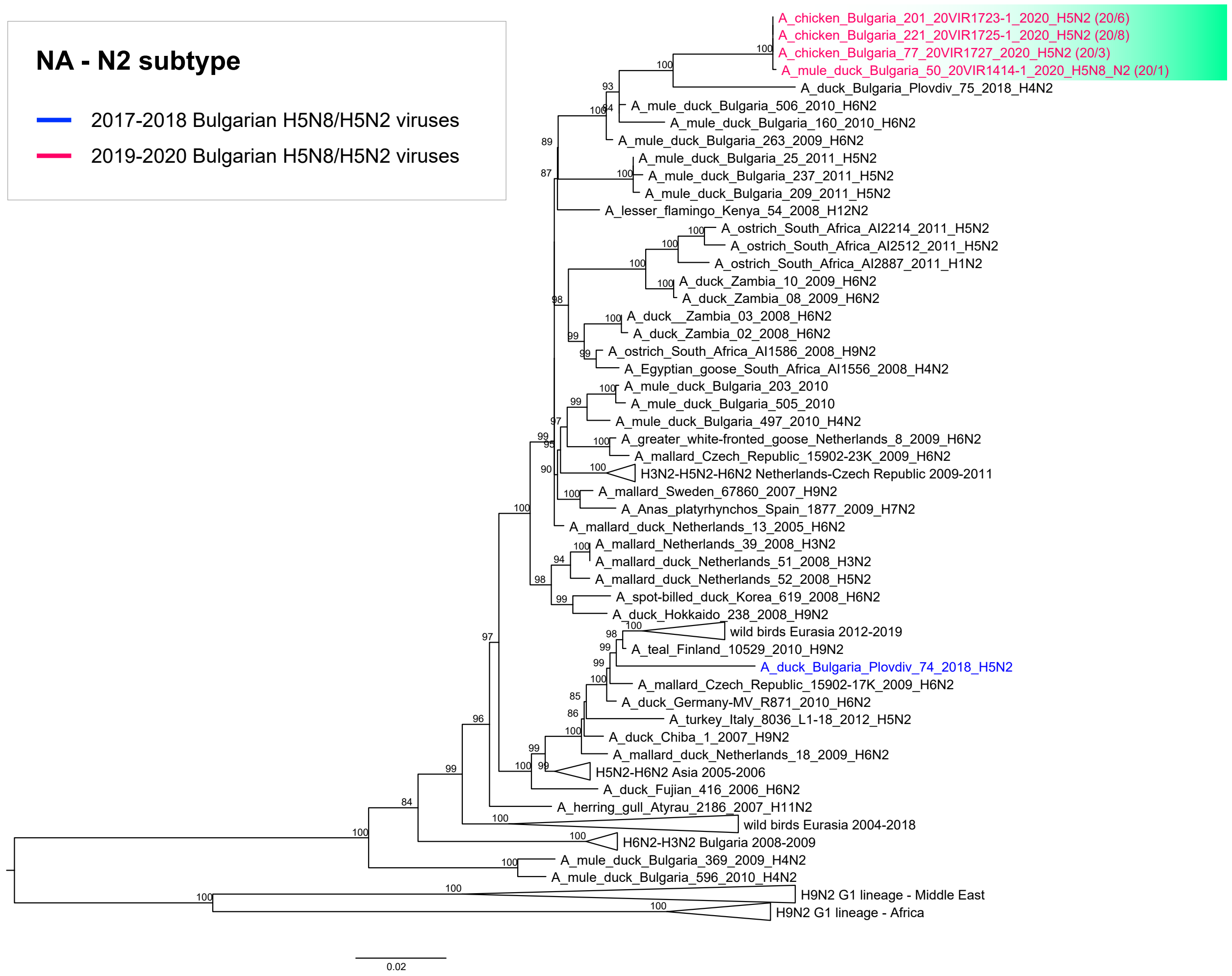

Supplementary Figure S5. Maximum Likelihood phylogenetic tree of the NA-N2 subtype gene. The tree was inferred using IQTREE v1.6.6. The light green box shows the cluster of 2020 Bulgarian H5N2 viruses. The viruses are coloured according to the years of collection. Ultrafast bootstrap supports higher than 80 are indicated next to the nodes.

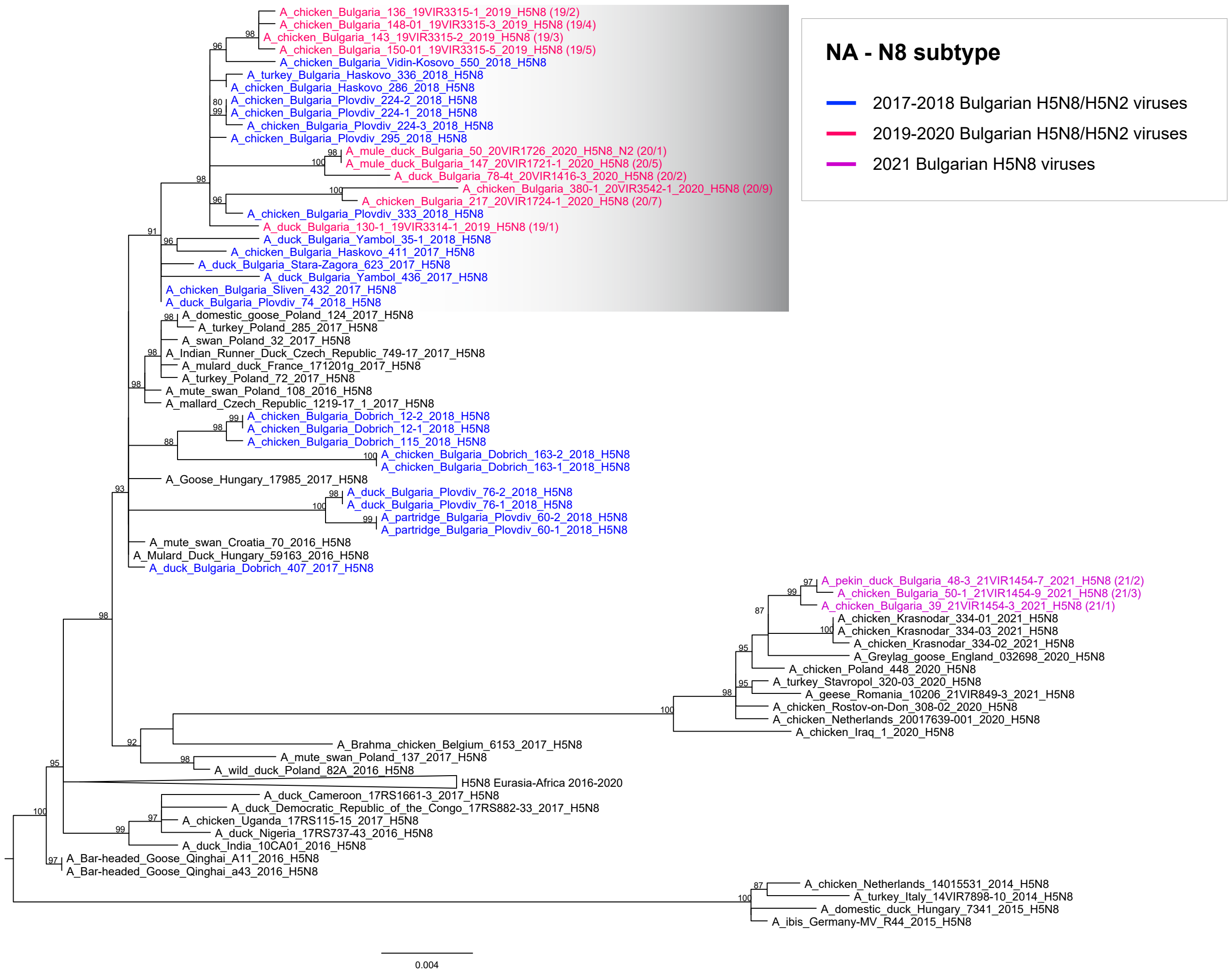

Supplementary Figure S6. Maximum Likelihood phylogenetic tree of the NA-N8 subtype gene. The tree was inferred using IQTREE v1.6.6. The grey box shows the cluster of Bulgarian H5 (N8 and N2) viruses identified in 2019-2020 together with some previously reported 2018 H5N8-H5N2 detections. The viruses are coloured according to the years of collection. Ultrafast bootstrap supports higher than 80 are indicated next to the nodes.

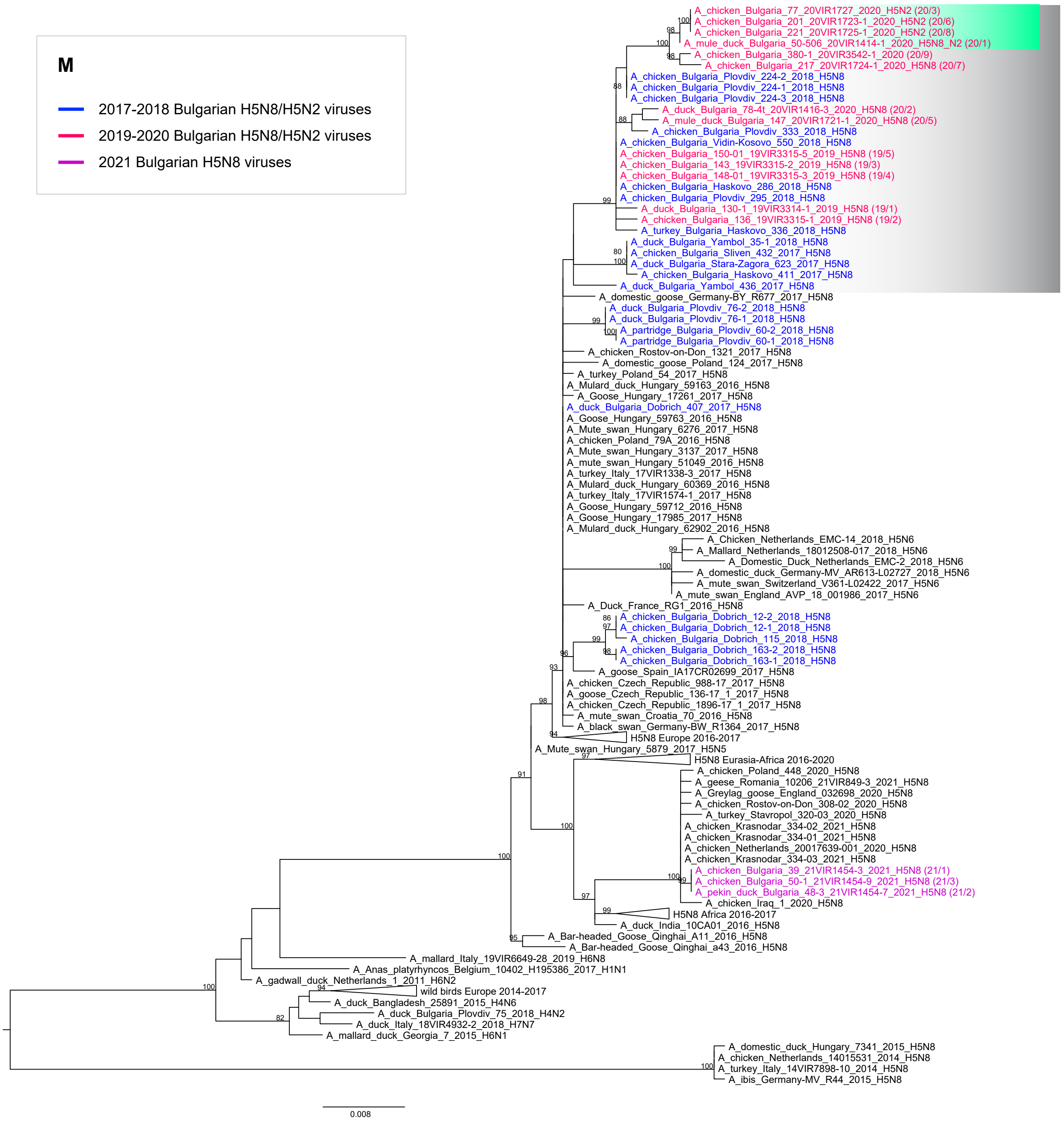

Supplementary Figure S7. Maximum Likelihood phylogenetic tree of the M gene. The tree was inferred using IQTREE v1.6.6. The grey box shows the cluster of Bulgarian H5 (N8 and N2) viruses identified in 2019-2020 together with some previously reported 2018 H5N8-H5N2 detections; the light green box shows the cluster of 2020 Bulgarian H5N2 viruses. The viruses are coloured according to the years of collection. Ultrafast bootstrap supports higher than 80 are indicated next to the nodes.

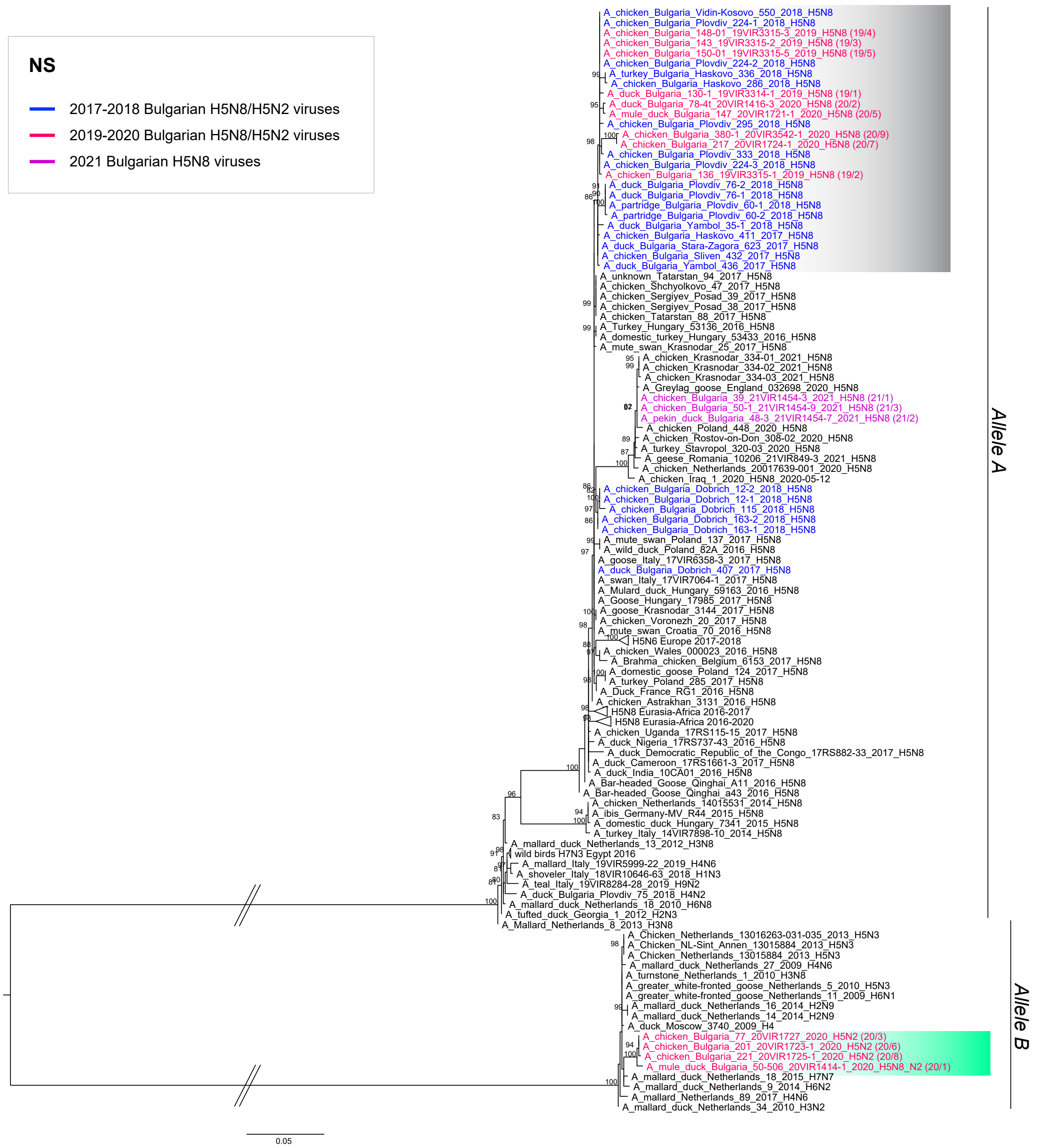

Supplementary Figure S8. Maximum Likelihood phylogenetic tree of the NS gene. The tree was inferred using IQTREE v1.6.6. The grey box shows the cluster of Bulgarian H5 (N8 and N2) viruses identified in 2019-2020 together with some previously reported 2018 H5N8-H5N2 detections; the light green box shows the cluster of 2020 Bulgarian H5N2 viruses. The viruses are coloured according to the years of collection. Ultrafast bootstrap supports higher than 80 are indicated next to the nodes.

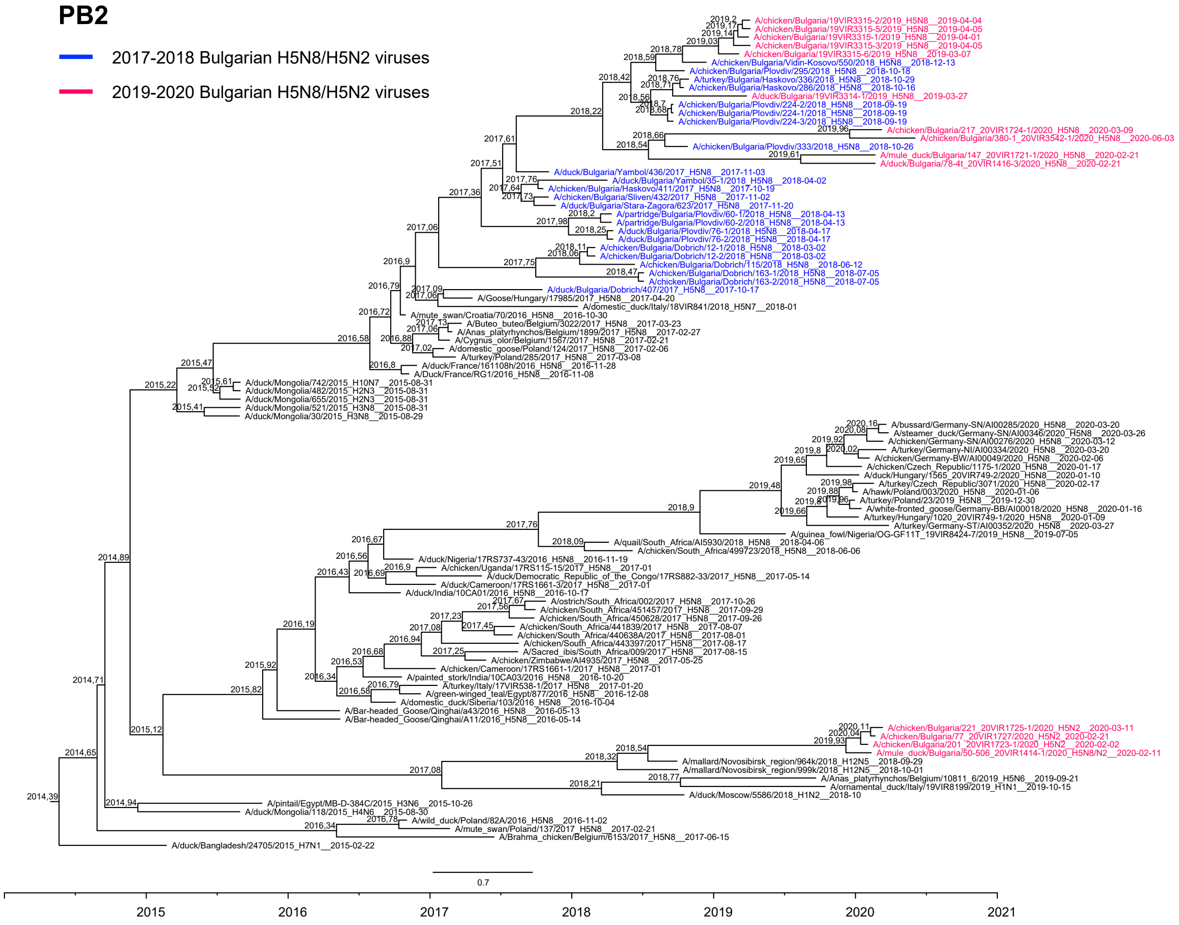

Supplementary Figure S9. Maximum Clade Credibility Tree of the PB2 gene. The tree was inferred using BEAST 1.10.4. The Bulgarian viruses are coloured according to the years of collection. A time scale axis is shown beneath the tree.

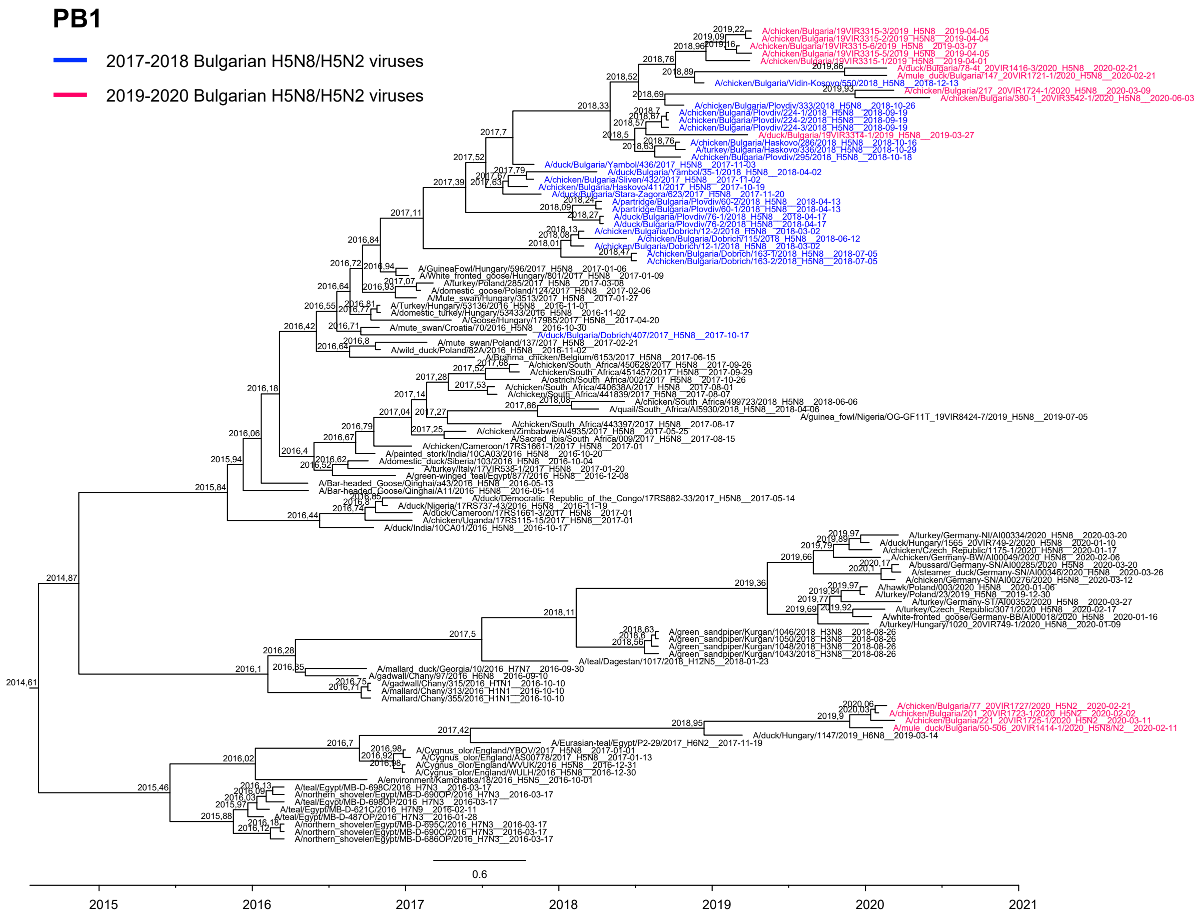

Supplementary Figure S10. Maximum Clade Credibility Tree of the PB1 gene. The tree was inferred using BEAST 1.10.4. The Bulgarian viruses are coloured according to the years of collection. A time scale axis is shown beneath the tree.

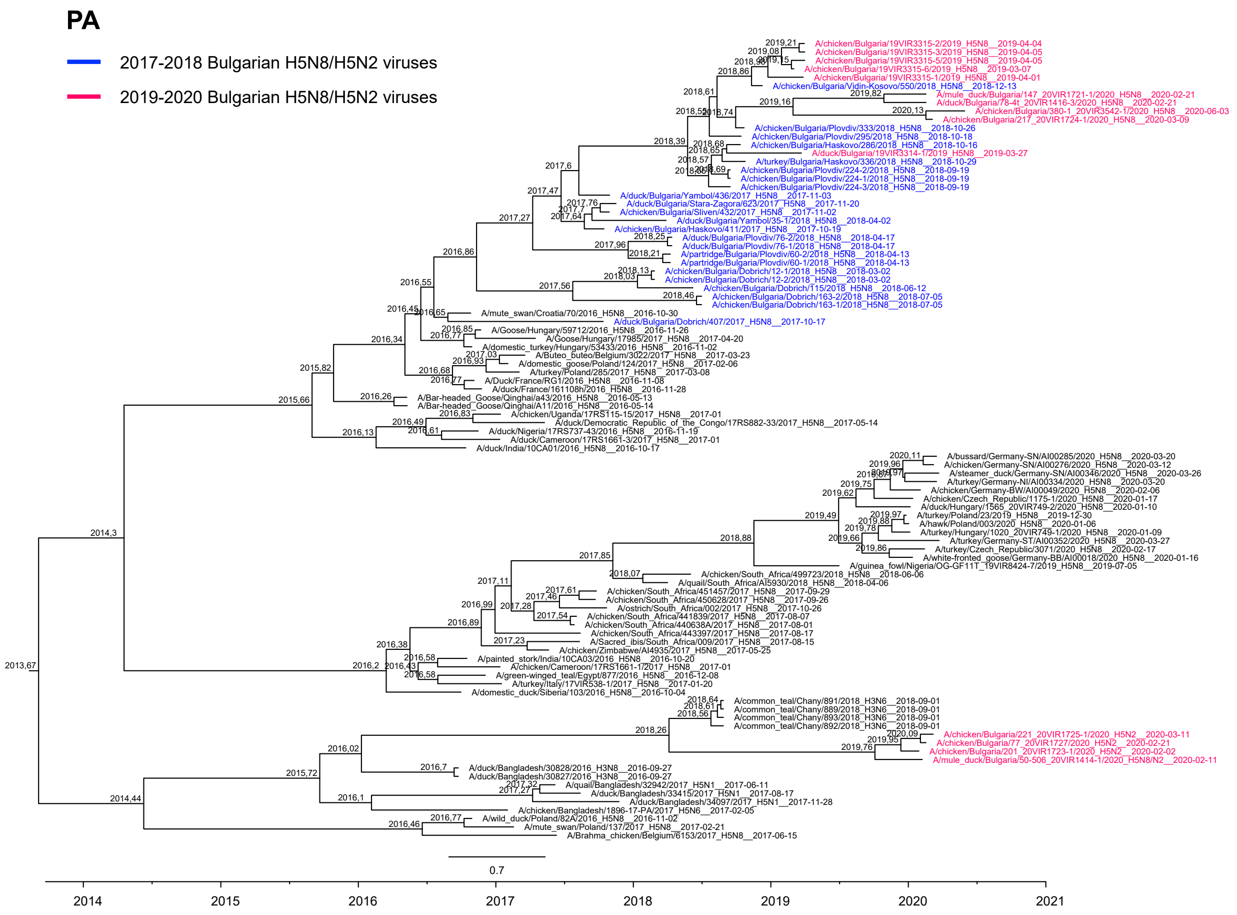

Supplementary Figure S11. Maximum Clade Credibility Tree of the PA gene. The tree was inferred using BEAST 1.10.4. The Bulgarian viruses are coloured according to the years of collection. A time scale axis is shown beneath the tree.

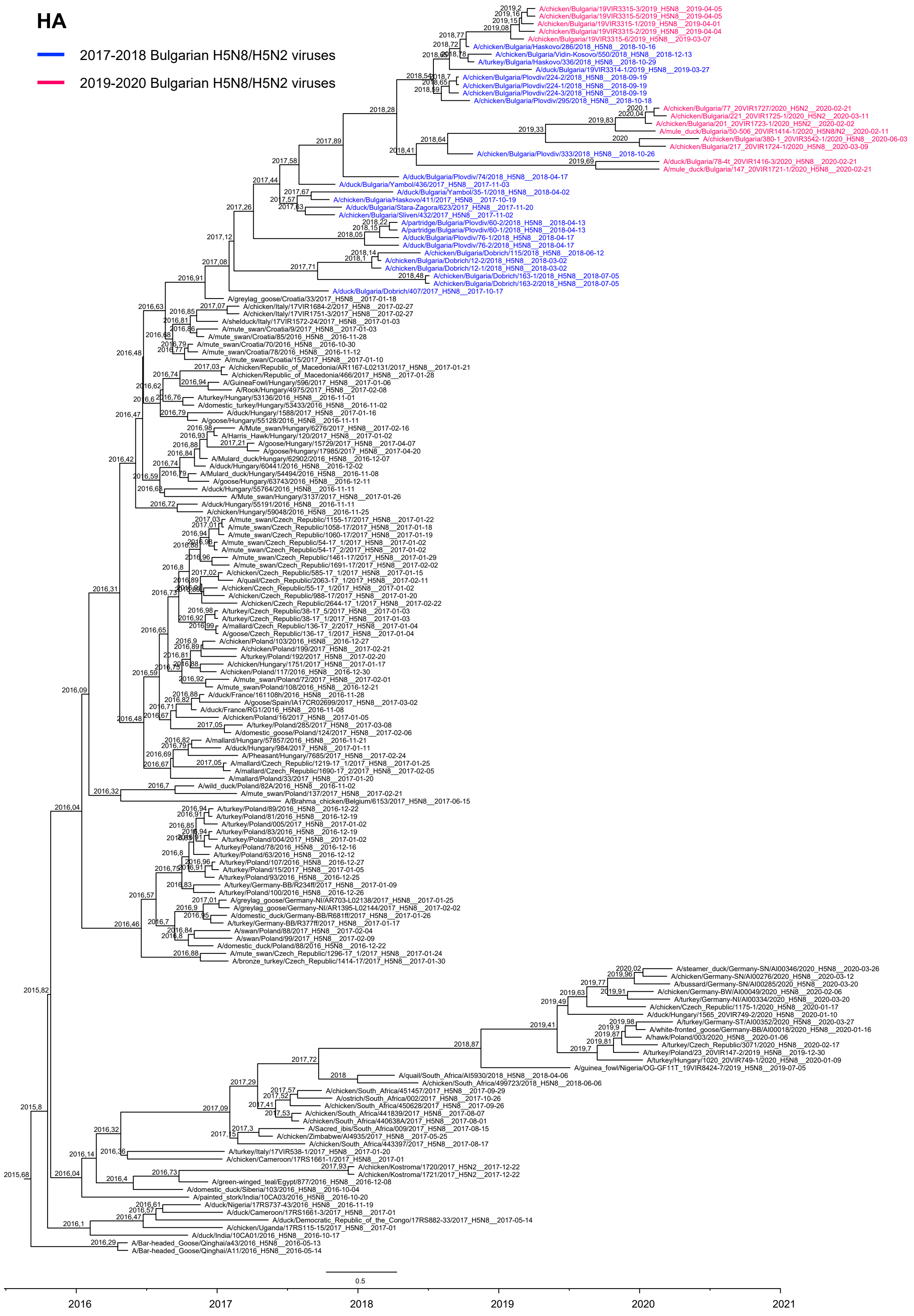

Supplementary Figure S12. Maximum Clade Credibility Tree of the HA gene. The tree was inferred using BEAST 1.10.4. The Bulgarian viruses are coloured according to the years of collection. A time scale axis is shown beneath the tree.

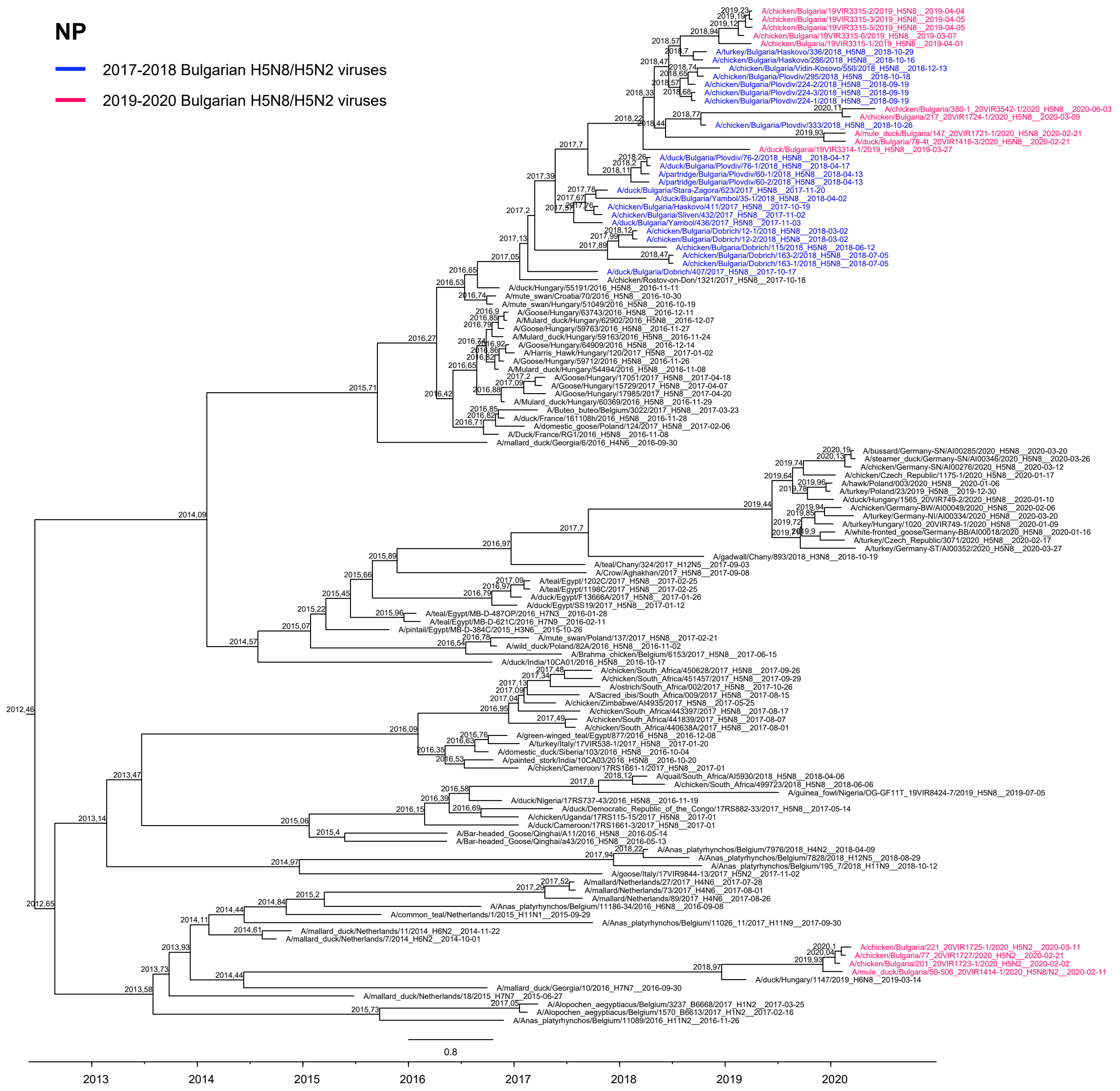

Supplementary Figure S13. Maximum Clade Credibility Tree of the NP gene. The tree was inferred using BEAST 1.10.4. The Bulgarian viruses are coloured according to the years of collection. A time scale axis is shown beneath the tree.

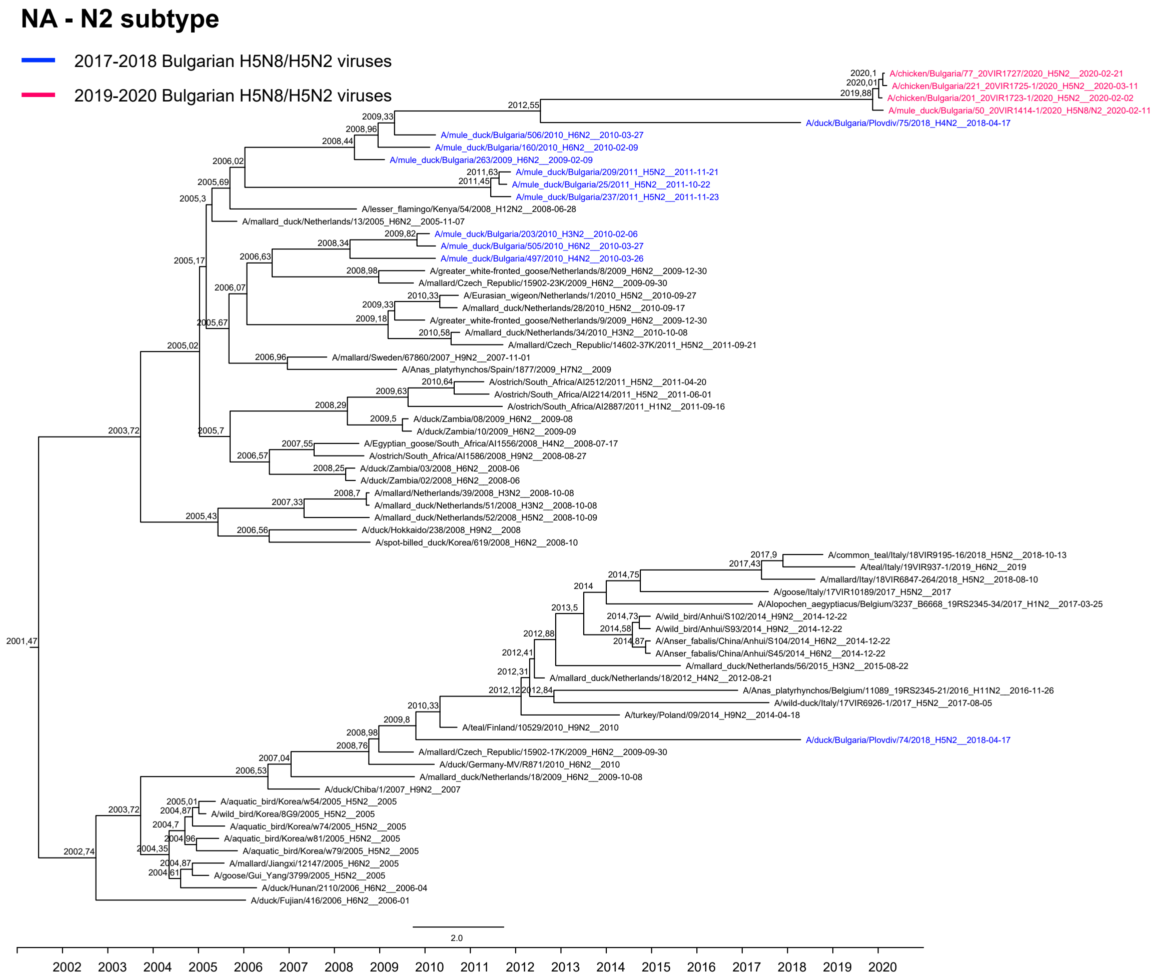

Supplementary Figure S14. Maximum Clade Credibility Tree of the NA-N2 subtype gene. The tree was inferred using BEAST 1.10.4. The Bulgarian viruses are coloured according to the years of collection. A time scale axis is shown beneath the tree.

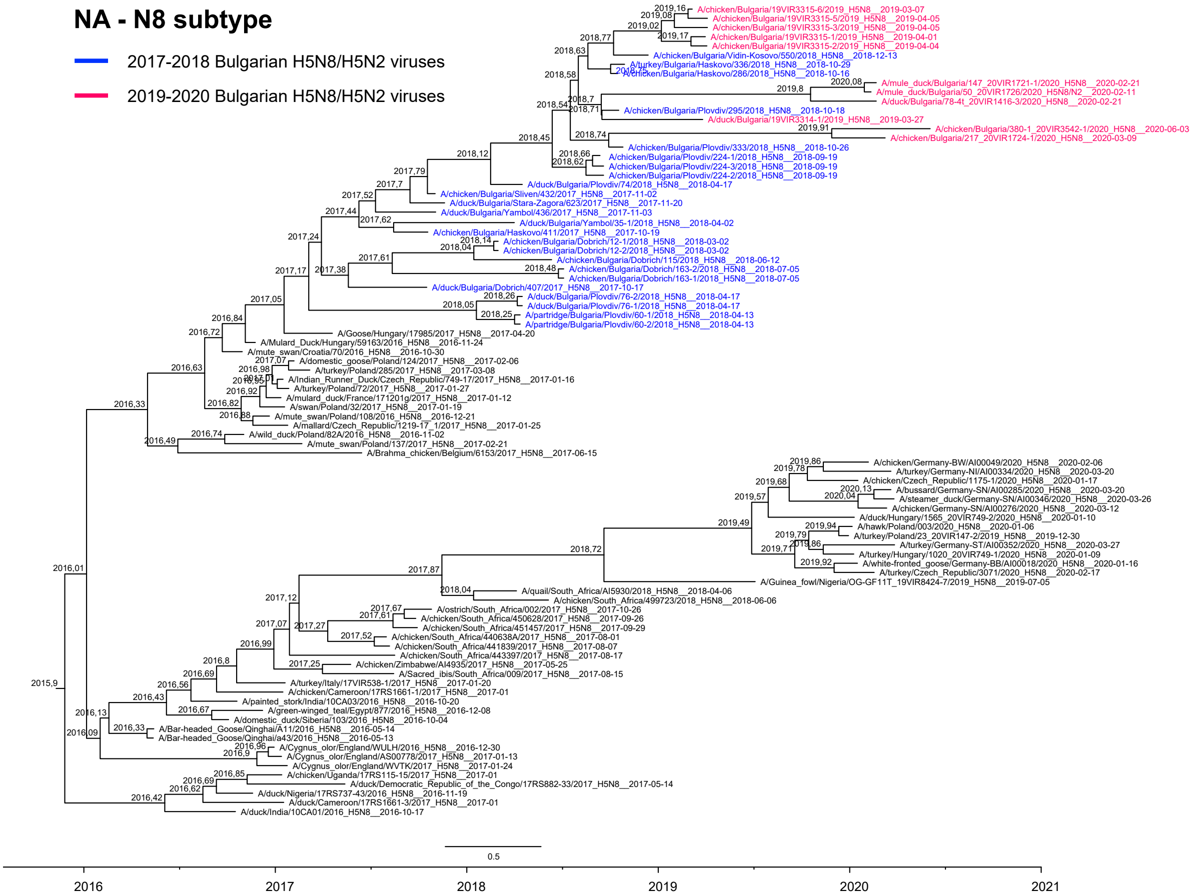

Supplementary Figure S15. Maximum Clade Credibility Tree of the NA-N8 subtype gene. The tree was inferred using BEAST 1.10.4. The Bulgarian viruses are coloured according to the years of collection. A time scale axis is shown beneath the tree.

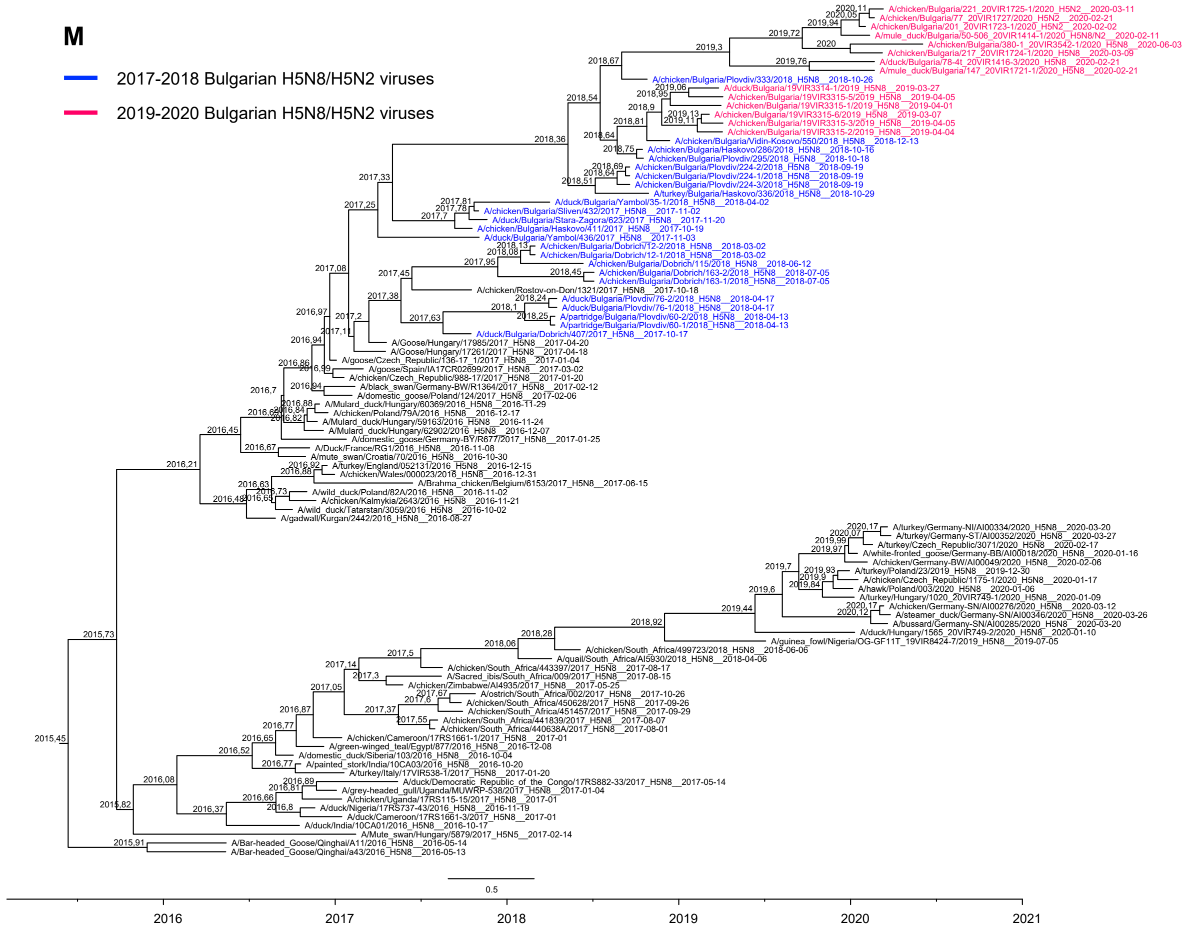

Supplementary Figure S16. Maximum Clade Credibility Tree of the M gene. The tree was inferred using BEAST 1.10.4. The Bulgarian viruses are coloured according to the years of collection. A time scale axis is shown beneath the tree.

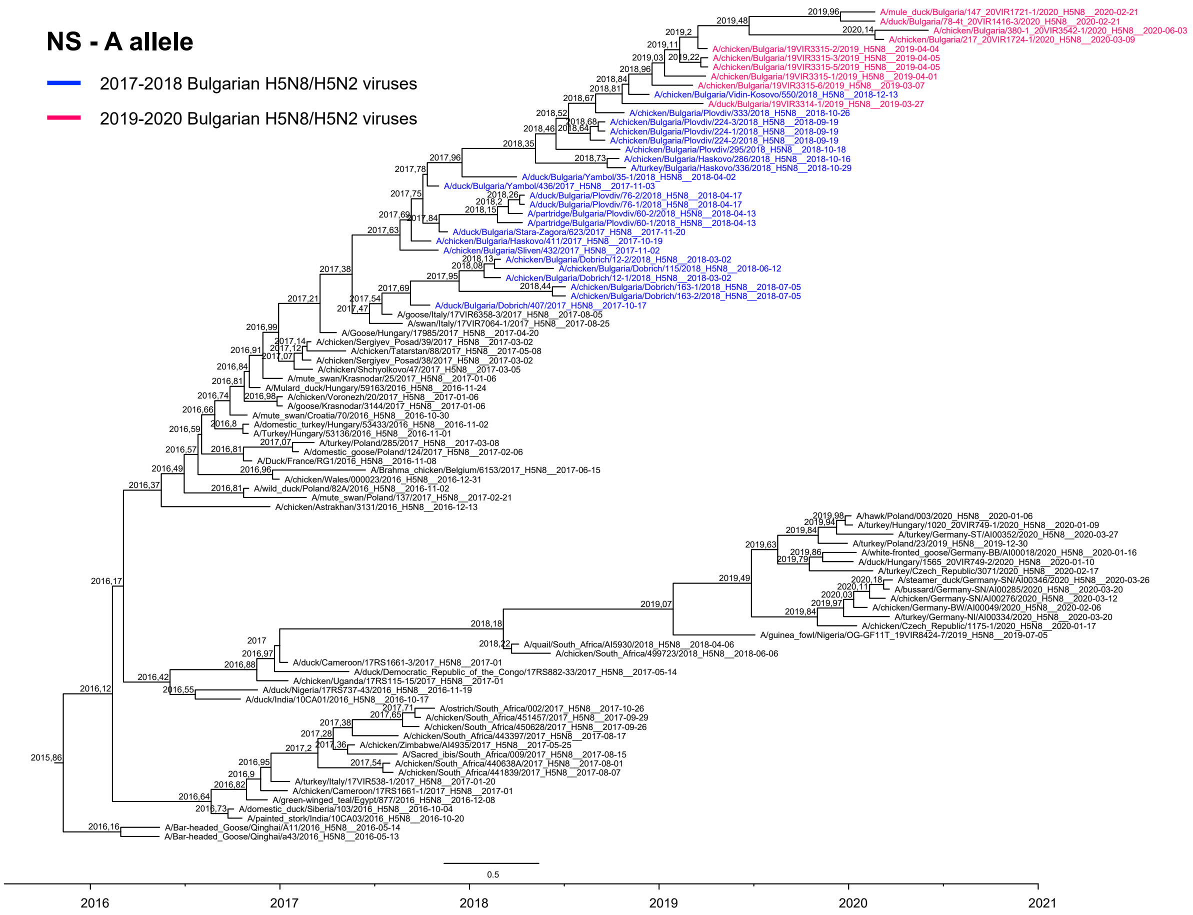

Supplementary Figure S17. Maximum Clade Credibility Tree of the NS (Allele A) gene. The tree was inferred using BEAST 1.10.4. The Bulgarian viruses are coloured according to the years of collection. A time scale axis is shown beneath the tree.

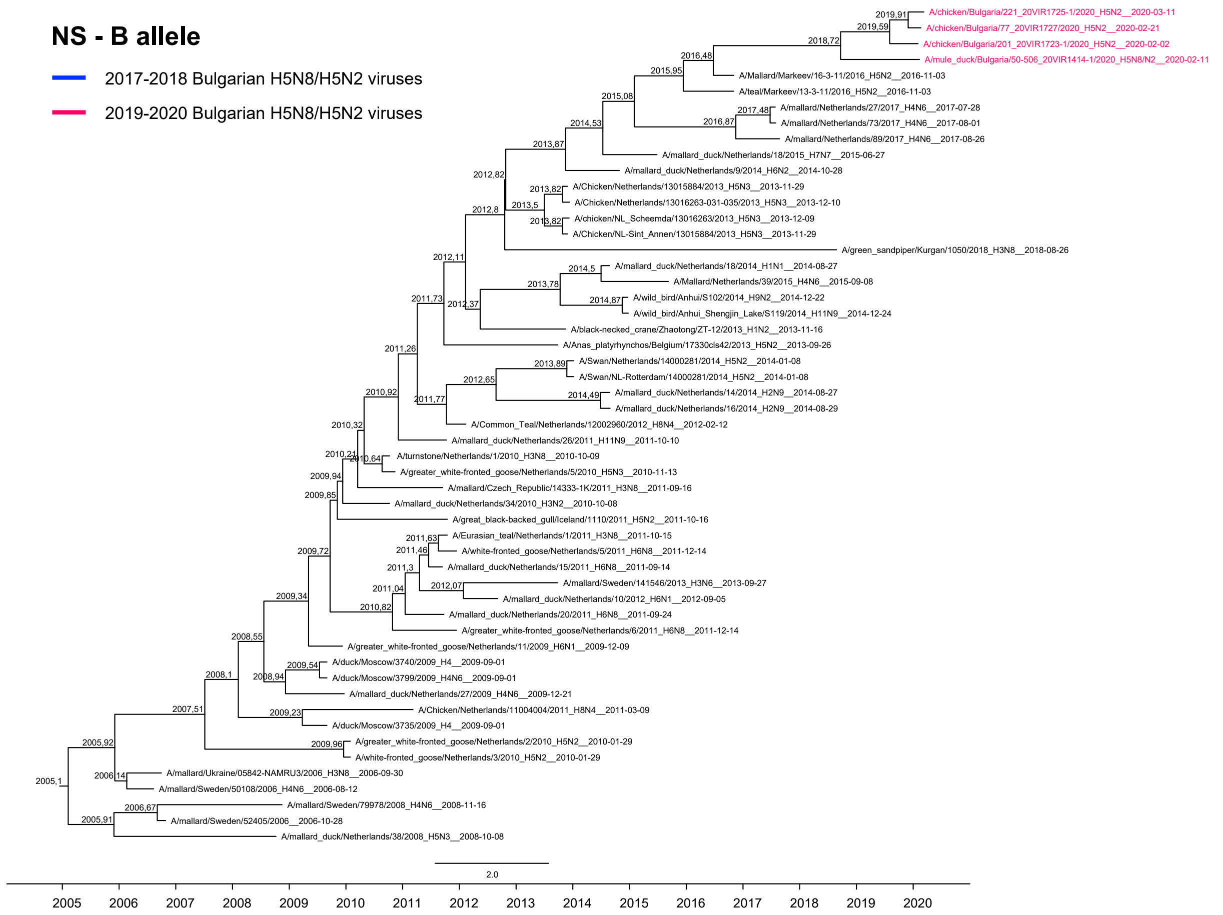

Supplementary Figure S18. Maximum Clade Credibility Tree of the NS (Allele B) gene. The tree was inferred using BEAST 1.10.4. The Bulgarian viruses are coloured according to the years of collection. A time scale axis is shown beneath the tree.
